# The Effect of Pet Insurance on Presurgical Euthanasia of Dogs With Gastric Dilatation-Volvulus: A Novel Approach to Quantifying Economic Euthanasia in Veterinary Emergency Medicine

**DOI:** 10.3389/fvets.2020.590615

**Published:** 2020-12-08

**Authors:** Manuel Boller, Tereza S. Nemanic, Jarryd D. Anthonisz, Magdoline Awad, Joshua Selinger, Elise M. Boller, Mark A. Stevenson

**Affiliations:** ^1^Faculty of Veterinary and Agricultural Sciences, Melbourne Veterinary School, University of Melbourne, Werribee, VIC, Australia; ^2^Faculty of Veterinary and Agricultural Sciences, Translational Research and Clinical Trials (TRACT), The University of Melbourne, Werribee, VIC, Australia; ^3^PetSure, Chatswood, NSW, Australia

**Keywords:** gastric dilatation-volvulus, insurance, veterinary, economic, euthanasia, dog, bloat

## Abstract

Euthanasia of companion animals in veterinary emergency medicine is a common cause of death. Euthanasia is economic when it is the consequence of the pet owner's inability to afford essential treatment while a viable medical alternative to euthanasia exists. Gastric dilatation-volvulus (GDV) is an acute life-threatening emergency condition of dogs; if left untreated, rapid death is highly likely. Surgical treatment leads to survival of around 80-90% of dogs; however, such treatment is costly. Therefore, pre-surgical euthanasia may be largely economically motivated. Having pet insurance, a financial instrument to reduce the burden of unforeseen veterinary medical costs on pet owners, would be expected to abolish the risk for pre-surgical economic euthanasia. We therefore aimed to determine whether pet insurance attenuates the risk of pre-surgical economic euthanasia in dogs with GDV. Non-referred dogs (*n* = 260) with GDV and known insurance status seen at 24 emergency clinics over a 2-year period were included. Relevant data (e.g., insurance status, age, comorbidities, outcome) were retrospectively extracted from a pet insurer's claim records (insured animals) or from electronic medical records of participating hospitals (non-insured animals). Forty-one percent of dogs (106 of 260 dogs) did not survive to hospital discharge; 82 (77%) of non-survivors died before surgery, all through euthanasia. The pre-surgical euthanasia rate was 10% in insured and 37% in non-insured dogs (*p* < 0.001). When adjusted for the effect of age, deposit size, comorbidities, and blood lactate concentration, the absence of insurance increased the odds of pre-surgical euthanasia by a factor of 7.4 (95% CI 2.0 to 37; *p* = 0.002). Of dogs undergoing surgery, 86% survived to hospital discharge. Overall, 80% of insured animals and 53% of non-insured animals survived to hospital discharge (*p* < 0.001). Thus, insurance was associated with a marked decrease in risk of pre-surgical euthanasia indicating that the cause of pre-surgical euthanasia of dogs with GDV is predominantly economic in nature. The rate of pre-surgical euthanasia in dogs with GDV may emerge as a suitable marker to quantify economic decision making of pet owners and to measure the impact of financial interventions aimed at mitigating economic duress associated with cost of veterinary emergency care.

## Introduction

The collective experience of veterinary professionals suggests that the euthanasia of companion animals in veterinary emergency medicine is often the consequence of the pet owner's inability to afford life-saving medical care. Such economic decision making, herein termed “economic euthanasia,” can lead to significant emotional and moral distress to the pet owner as well as to the veterinary professionals involved in the euthanasia (1–5). Despite the magnitude of the emotional issues associated with economic euthanasia, details of its frequency and predisposing factors remain insufficiently studied and adequate systematic evaluation of financial interventions to attenuate the issue is lacking. Financial instruments that may assist pet owners to cope with unexpected veterinary expenditure include payment plans, loans, pet health insurance or support by charitable organizations (6). It seems intuitive to believe that pet insurance covering the costs of treatment for a life-threatening condition would lead to higher rates of such treatment and consequently reduce animal loss. However, the quantitative impact of pet health insurance on rates of euthanasia of companion animals in general, and specifically in the emergency setting, constitutes an important knowledge gap.

Gastric dilatation-volvulus (GDV) is an acute life-threatening disease of dogs affecting multiple body systems (7). The condition results in rapid distension of the stomach causing compression of the major abdominal blood vessels, impeding the return of venous blood to the heart, compromising cardiac output and leading to circulatory shock (7, 8). If the animal is left untreated, death is highly likely, but the population-wide survival rate of dogs treated surgically is relatively high. A large epidemiological study comprised of 492 emergency GDV cases across the UK demonstrated a survival to discharge rate of 79% in cases where surgery was carried out (9). Studies from the US found similar survival rates in animals undergoing surgery [>90% (10), 88% (11) and 84% (12, 13)] and comparable findings have been identified in a recent Australian study (87%) (14). The two most recent studies, including 498 and 736 cases of GDV, report that 64 and 82%, respectively, of all deaths of animals presenting with GDV occurred due to humane euthanasia prior to surgery (13, 14).

We propose a role for using euthanasia rates of dogs with GDV prior to surgery as a surrogate marker for economic decision making, as GDV in dogs combines the following general properties: (1) a relatively low cost of diagnosis; (2) a high likelihood of suffering and death without surgical treatment; (3) a high likelihood of survival and well-being if treated; (4) a high cost associated with treatment; (5) common condition of dogs globally allowing study in diverse socioeconomic environments. Notably, in many cases the reasons for the decision to euthanize prior to treatment are likely a combination of financial limitations, severity of illness, advanced age, and pre-existing comorbidities affecting quality of life, i.e., reasons that are not solely economic in nature (13). For proof of concept, we previously studied the effect of a financial instrument, pet insurance, to alleviate euthanasia prior to surgery in non-referred dogs with GDV presented to our emergency service. We found that such euthanasia was significantly reduced in insured compared to non-insured dogs, even when adjusted for lactate, age, and comorbidities (15).

In this study, we set out to investigate to what extent pre-surgical euthanasia of non-referred dogs with GDV is economically motivated. If economics play a significant role, then the presence of a model financial intervention, such as pet insurance, would be associated with a clear reduction in pre-treatment euthanasia.

The specific aim of our study was to determine the impact of a financial tool, in this case pet health insurance, on the euthanasia rate of non-referred dogs with GDV presenting to veterinary emergency practices in Southeast Australia. We hypothesized that the presence of pet insurance compared to no insurance significantly reduces the risk of pre-surgery euthanasia of dogs with GDV. The results of this study are an important step toward an objective understanding of the problem of economic euthanasia in emergency veterinary medicine, and to establish a quantitative understanding on its role as a cause of preventable death.

## Materials and Methods

### Study Population

The study was designed as a case-control study. The population of interest comprised non-referred dogs with radiographically confirmed GDV presented to emergency hospitals in Southeast Australia between 1 January 2017 and 31 December 2018. Dogs were classified as cases if they were euthanized pre-treatment, and controls if they underwent surgical treatment. Details of exposure-positive (i.e., insured) dogs for this study were extracted from a pet insurance data base (PetSure Pty Ltd, Castle Hill, NSW, Australia). Data for non-insured animals were obtained from electronic medical records (EMR) of emergency hospitals that agreed to contribute to the study in Victoria (VIC) and New South Wales (NSW). Dogs were excluded from the study if they were referred from or to another veterinary practice or if no abdominal radiographs were taken; we limited the study to non-referred cases because owners that are referred are typically already committed and aware of the economic aspects of care.

### Insurance Status

Misclassification of exposure status for exposure-positive (insured) dogs was unlikely due to the data originating from a pet insurer's database. To reduce the probability of misclassification of exposure status for exposure-negative (uninsured) dogs we required a clear statement to be present in the EMR about the absence of insurance at the time of GDV. Dogs with no mention of the absence of insurance status were excluded from the study.

### Data Collection

Details of cases of GDV in VIC and NSW for insured dogs for the 2-year study period were provided by the pet insurer. The insurer's database contains details of insurance claims submitted by pet owners and details of the attending veterinarian. Claim details are reviewed by a veterinarian or veterinary nurse employed by the insurer and entered into the insurer's database according to company-specific operational definitions. Cases were identified as claims with GDV as a labeled diagnostic description, and data required for this study were extracted by the company data scientist and bulk uploaded to an electronic study database built in REDCap (16).

Seven emergency hospitals agreed to visitation of study team members for data extraction of non-insured animals from their EMR. Two members of the research team (TSN, JDA) visited each hospital to gather details of non-insured GDV cases. We used the terms “dog” and keywords “GDV,” “gastric dilation,” “gastric dilatation,” “volvulus,” “torsion,” and “gastropexy” to search each practice's EMR database to identify GDV cases. We manually reviewed the records of individual animals for inclusion and exclusion criteria. We then extracted relevant data elements and entered them into the study's electronic database.

### Data Characteristics and Outcome Measures

Data recorded in the research database (Appendices 1, 2) included information about the hospital to which the animal was presented, demographic details of the animal itself, clinical metrics recorded at the time of presentation and an unambiguous statement about the presence or absence of euthanasia.

Hospital data comprised the type of practice (i.e., general practitioner, or referral practice), the extent of emergency services provided and details of payment options available to pet owners. We recorded whether a deposit was required at admission, the size of the deposit in proportion to the estimate, and whether payment plans or other finance options were available. In insured cases, for privacy-related reasons, this information was obtained by the insurer via direct contact with hospitals; likewise, our study personnel contacted hospitals with non-insured cases to obtain these data.

Animal biographical data comprised static details (i.e., breed, age, sex), comorbidities, insurance and referral status. For insured dogs, we recorded the presence of a comorbidity if the insurance database contained a diagnosis of chronic diseases preceding the GDV claim. In non-insured dogs, we extracted comorbidity information from the EMR held by the practice where the GDV dog was presented. We scrutinized records for referral status, as only non-referred cases were included in the study. For insured dogs, the presence of a claim at another practice with a coded diagnosis commensurate with GDV shortly preceding the claim from the practice with the GDV episode of interest was defined as a referred case and therefore excluded. For non-insured dogs, an animal was considered referred based on respective notes in EMR history or referral communication.

Data related to the GDV event included diagnostic measures and blood lactate concentration at presentation. For insured dogs, GDV was defined as the presence of a diagnostic code for GDV combined with a record that abdominal radiographs were taken at the time of presentation. For non-insured dogs, GDV was defined as the presence of an appropriate history and clinical signs in combination with abdominal radiographs to support the diagnosis, and the clinician's diagnosis of GDV in the EMR. The first lactate concentration determined after presentation but before surgery was recorded. This was accomplished by manual review of medical records of both insured and non-insured dogs.

The outcome for this study was the presence or absence of euthanasia prior to surgery. In insured dogs, this was identified as a dog with a GDV diagnosis as outlined above, and a claim for a charge for consultation and euthanasia but not for surgery. In non-insured cases, we arrived at this outcome when a diagnosis for GDV was made but the animal did not proceed to surgery and a charge for euthanasia was present. For dogs that were not euthanized, we recorded whether the dog survived to hospital discharge. For dogs that had a surgical charge but did not survive, we further noted whether this death occurred due to cardiopulmonary arrest (CPA) or euthanasia based on insurance database charges or EMR information.

### Data Analysis

#### Univariate Analyses

Unconditional associations between each of the patient characteristics that were hypothesized to influence the risk of euthanasia were computed using the Mann-Whitney U test for continuous data and the odds ratio for categorical data. Continuous variables included the age of the dog at the time of presentation (in years) and blood lactate concentration (in mmol/L). Categorical explanatory variables included insurance status, requirement for > 50% of the total estimated fee as a deposit before surgery, availability of payment plans, hospital location (VIC, NSW), calendar year (2017, 2018), breed (mixed or pure-bred), sex (male, female), age category (non-geriatric, geriatric) and the presence of at least one comorbidity. Animals were classified as “young” or “geriatric” based on reported median longevity for each breed (17, 18). Lactate concentration was dichotomized as low (i.e., value <6.0 mmol/L), or high (i.e., value ≥ 6.0 mmol/L) as this cut-off was previously shown to be associated with GDV survival (19). Explanatory variables with an unconditional association with euthanasia status at *p* < 0.20 using the chi-squared test were selected for multivariable modeling.

#### Multivariate Analyses

A binary logistic regression model was developed where the probability of euthanasia before surgery was parametrized as a function of the explanatory variables with *p* < 0.20 identified in the univariate analyses. Explanatory variables that were not statistically significant were removed from the logistic regression model one at a time; beginning with least significant until the estimated regression coefficients for all explanatory variables retained were significant at *p* < 0.05 based on the likelihood ratio test. Explanatory variables that were excluded from the initial model during this stage were tested for inclusion in the final model and were retained if any changed the regression coefficients by >20%.

## Results

### Study Population

**Figure 1:**
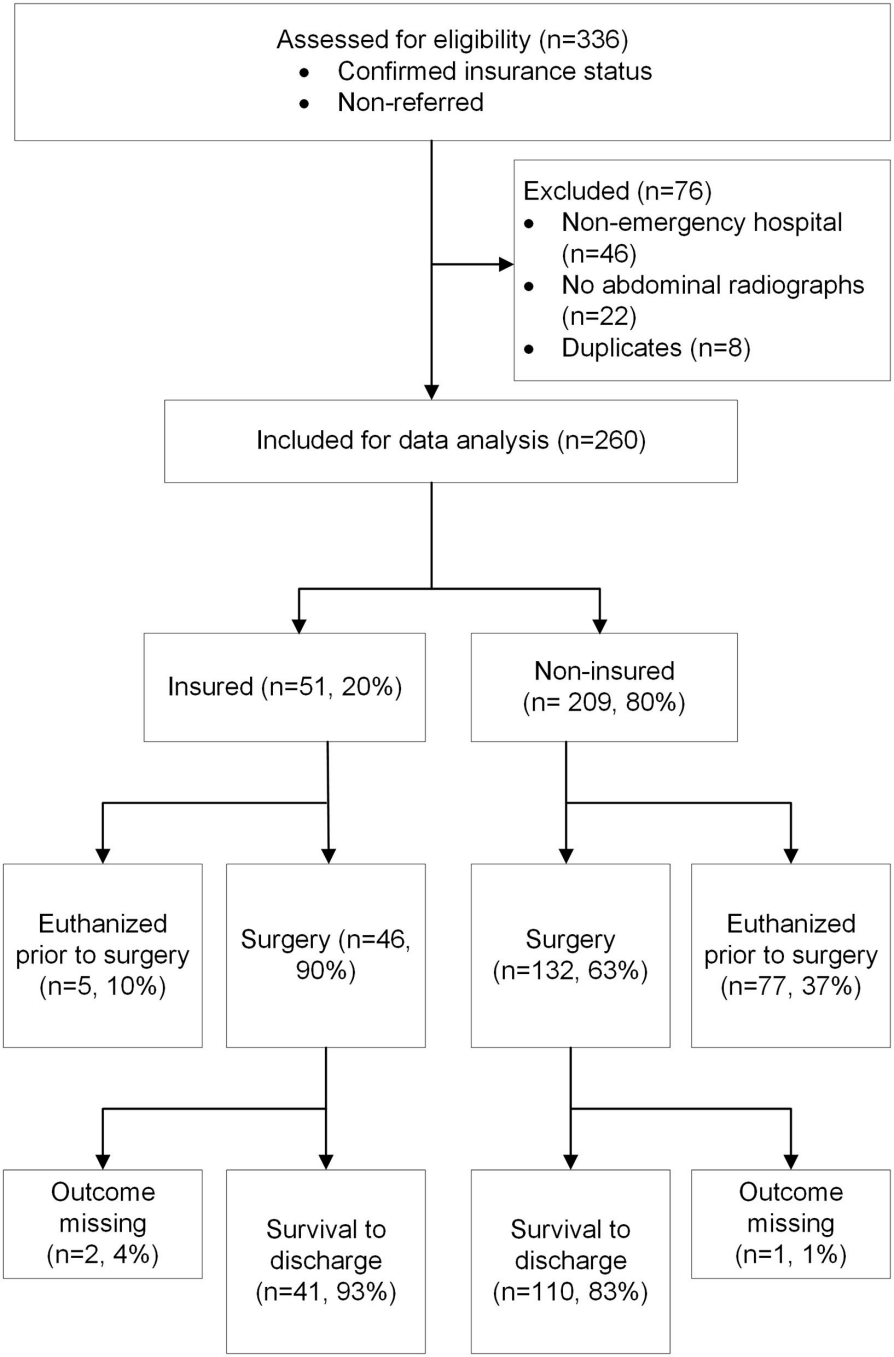
Flow diagram summarizing the study case selection process and the observed outcome of dogs presented to emergency clinics for gastric dilatation-volvulus.

Records from 336 dogs with presumptive GDV that were non-referred and had confirmed insurance status were identified from hospital medical records and from the insurer's claims database within the imposed time frame (Figure 1). From these, records were excluded if the animal was presented to a non-emergency hospital (*n* = 46, 14%), if no abdominal radiographs were taken for confirmation of GDV (*n* = 22, 7%), and if duplicate entries as per matching of hospital post code, date of GDV, sex and breed were detected (*n* = 8, 2%). Data from 260 non-referred dogs with known insurance status and presenting to 24 emergency clinics with radiographically confirmed GDV were available for analysis. The mean age of dogs at the time of presentation was 8 years (range: 0.3 to 16 years), dogs were predominantly purebred (*n* = 219, 84%; 95% CI, 79 to 88%) and were more often male than female (*n* = 163, 67%; 95% CI, 61 to 73%). Most of the animals were seen in emergency clinics or services open 24 h (*n* = 196, 75%; 95% CI, 70 to 80%), followed by emergency/critical care centers with at least one board-certified emergency clinician (*n* = 45, 17%; 95% CI, 13 to 22%) and emergency clinics or services open after regular business hours only (*n* = 19, 7%; 95% CI, 5 to 11%). The majority of dogs were presented to hospitals in the state of Victoria (*n* = 229, 88%; 95% CI, 84 to 92%) whilst the remainder were seen in New South Wales. The dataset comprised 209 uninsured (80%; 95% CI, 75 to 85%) and 51 insured (20%; 95% CI, 15 to 25%) dogs.

### Economic Characteristics

All of the dogs in this study were treated in hospitals requiring advanced payment of a percentage of the total estimated cost (i.e., a deposit), with the deposit requirement applying to both non-insured and insured dogs. All but one dog were seen in hospitals that allowed payment via an external financing service (i.e., VetPay). Eleven dogs (4%; 95% CI, 2 to 7%) were presented to hospitals allowing payment plans, 10 of which had insurance and one did not.

### Mortality Prior to Surgery and Its Risk Factors

**Figure 2:**
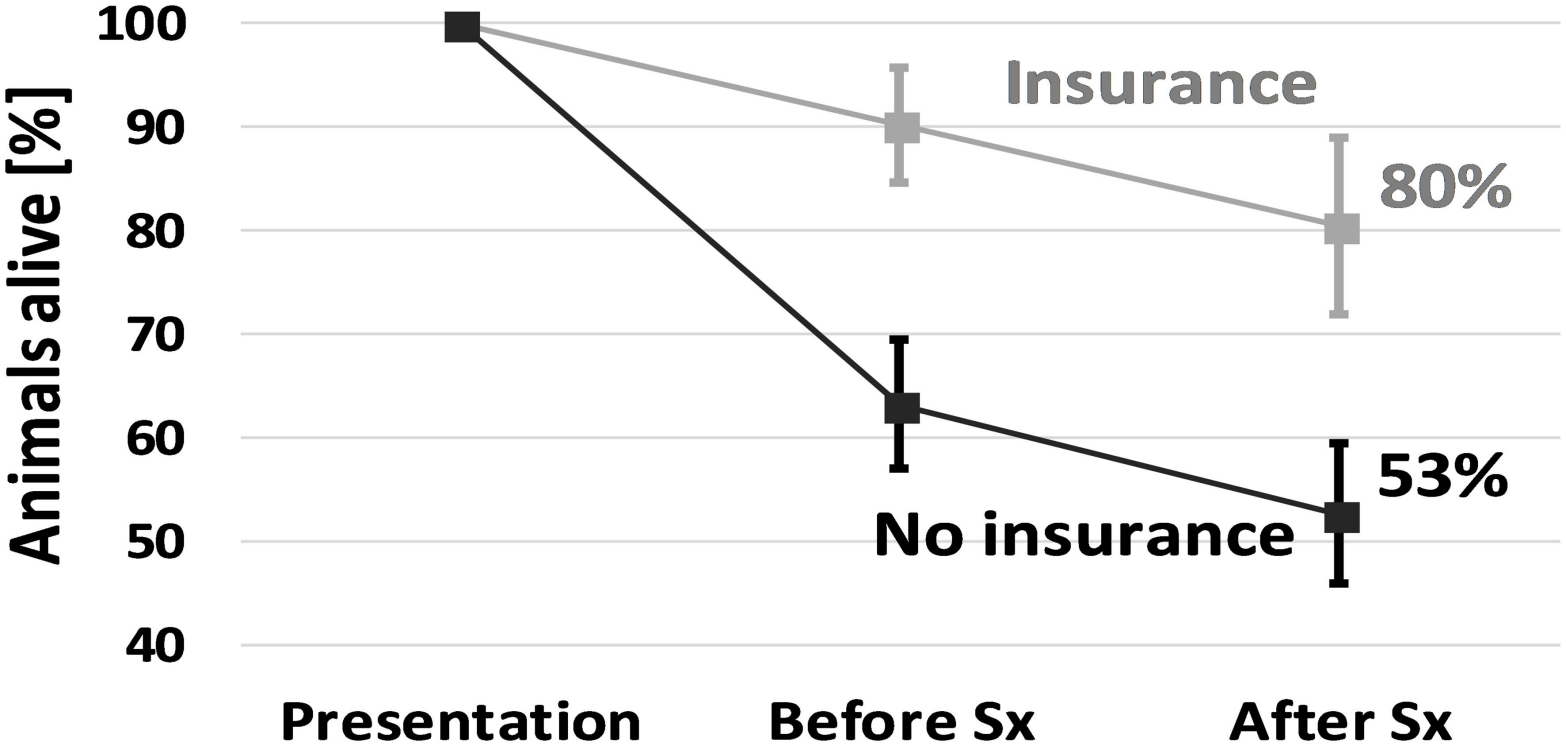
Animals alive at different stages during hospitalization from presentation, before surgery (“Before Sx”) to hospital discharge (“After Sx”). A larger proportion of non-insured animals died in the period prior to surgery compared to those that were insured, while the incremental rate of death over the surgical period was similar. Differing pre-surgical euthanasia rates were responsible for the large variation in survival of dogs with GDV. Error bars indicate 95% confidence intervals.

Almost one third of the dogs (*n* = 82, 32%; 95% CI, 26 to 37%) died prior to surgery, all through humane euthanasia and none through CPA. Lactate concentrations at presentation were not recorded in 91 dogs (35%), but when available, they were significantly higher in animals euthanized prior to surgery compared to those that were not (Table 1). Of note, the odds for euthanasia prior to surgery were significantly higher in dogs in which no lactate was measured at presentation, whether the lactate concentration was <6.0 mmol/L (OR, 11; 95% CI, 5.2 to 23) or ≥ 6.0 mmol/L (OR, 3.3; 95% CI, 1.7 to 6.6) (Table 2). Age was higher in animals euthanized prior to surgery compared to those not euthanized (Table 1). Similarly, animals denoted geriatric based on breed-specific longevity data were of higher risk for euthanasia prior to surgery (Table 2). A higher percentage of non-insured (37%; 95% CI, 31 to 44%) than insured animals (10%; 95% CI, 4 to 21%) were euthanized prior to surgery (*p* < 0.001) (Figure 2), and non-insurance was a significant risk for euthanasia prior to surgery (OR, 5.4; 95% CI, 2.0 to 14) (Table 2). Additional factors associated with a higher risk for euthanasia prior to surgery included a requirement for a higher deposit (OR, 2.9; 95% CI, 1.3 to 6.9), and female sex (OR, 1.8; 95% CI, 1.0 to 3.2) (Table 2). None of the other variables assessed as risk factors (i.e., state, case year, breed type, and comorbidities, payment plans) demonstrated a statistically significant association with the occurrence of euthanasia prior to surgery.

**Table 1:** Descriptive statistics of each of the continuous variables assessed for an association with euthanasia of dogs presenting to emergency clinics with GDV.

**Variable**	***n***	**Mean (SD)**	**Median (Q1, Q3)**	**Min, max**	**Missing**
**LACTATE (MMOL/L)^a^**					
Euthanasia +	29	7.4 (3.1)	6.9 (4.9, 9.5)^b^	3.4, 15.0	53
Euthanasia –	111	4.8 (2.9)	4.1 (2.7, 6.6)	0.6, 12.5	67
Total	140	5.3 (3.1)	4.5 (2.9, 6.8)	0.6, 15.0	120
**AGE (YEARS)^a^**					
Euthanasia +	82	9.4 (3.4)	10 (6, 11)^b^	2, 16	0
Euthanasia –	178	7.3 (3.2)	7 (4, 9)	1, 16	0
Total	260	8.0 (3.4)	8 (5, 9)	1, 16	0

a *Mann-Whitney U-test*.

b *p < 0.0001*.

**Table 2:** Unconditional associations between pre-surgical euthanasia status and each of the explanatory variables included in this study.

**Risk factor**	**Euthanized**			**Risk^a^**	**OR (95% CI)**	***p* value^b^**
	**Yes**	**No**	**Total**			
**Insurance status**						
No	77	132	209	36.8	5.4 (2.0 to 14)	<0.0001
Yes	5	46	51	9.8	Reference	
Total	82	178	260			
**Deposit (percent of quote)**						
≥50%	75	138	213	35.2	2.9 (1.3 to 6.9)	0.0128
<50%	7	38	45	15.6	Reference	
Missing	0	2	2			
Total	82	178	260			
**Payment plan available**						
No	80	167	247	32.3	1.8 (0.5 to 6.3)	0.2987
Yes	2	9	11	18.2	Reference	
Missing	0	2	2			
Total	82	178	260			
**State**						
VIC	75	154	229	32.8	1.7 (0.70 to 4.0)	0.3066
NSW	7	24	31	22.6	Reference	
Total	82	178	260			
**Year**						
2018	50	99	149	33.6	1.2 (0.73 to 2.1)	0.5001
2017	32	79	111	28.8	Reference	
Total	82	178	260			
**Breed**						
Mixed	15	26	41	36.6	1.3 (0.65 to 2.6)	0.4671
Purebred	67	152	219	30.6	Reference	
Total	82	178	260			
**Sex**						
Female	33	47	80	41.3	1.8 (1.0 to 3.2)	0.0405
Male	45	118	163	27.6	Reference	
Missing	4	13	17			
Total	82	178	260			
**Age category**						
Geriatric	45	38	83	54.2	4.5 (2.5 to 7.9)	<0.0001
Non-geriatric	37	140	177	20.9	Reference	
Total	82	178	260			
**Comorbidities**						
Yes	24	48	72	33.3	1.1 (0.62 to 2.0)	0.7762
Not recorded	58	129	187	31.0	Reference	
Missing	0	1	1			
Total	82	178	260			
**Lactate**						
Not recorded	52	39	91	57.1	11 (5.2 to 23)	<0.0001
High	18	44	62	29.0	3.2 (1.7 to 6.6)	0.004
Low	12	95	107	11.2	Reference	
Total	82	178	260			

a *Number of dogs euthanized per 100 dogs at risk*.

b *Likelihood ratio test*.

After adjusting for the combined effect of insurance status, deposit size, age category, sex and lactate group, the absence of insurance at the time of GDV increased the odds of euthanasia prior to surgery by a factor of 7.4 (95% CI, 2.0 to 37) (Table 3). A clear effect of age on the risk of euthanasia prior to surgery was present, with geriatric patients more likely to be euthanized prior to surgery (OR, 4.4; 95% CI, 2.1 to 9.3) compared with younger patients. Patients that had no lactate recorded had increased odds of euthanasia (OR, 15; 95% CI, 6.7 to 37) compared with patients with low lactate.

**Table 3:** Regression coefficients and their standard errors from a logistic regression analysis of factors associated with the risk of pre-surgical euthanasia of dogs with GDV.

**Variable**	**Coefficient (SE)**	***p* value**	**OR**	**95% CI**	
				**Lower**	**Upper**
Intercept	1.28 (0.36)	0.0003			
**INSURANCE STATUS**					
Non-insured	1.00 (0.37)	0.0022	7.4^a^	2.0	37
Insured	Reference	-	1.0		
**DEPOSIT REQUIRED**					
≥50% of estimate	−0.01 (0.46)	0.9837	1.0	0.16	6.4
<50% of estimate	Reference	-	1.0		
**SEX**					
Female	0.31 (0.18)	0.0902	1.8	0.91	3.8
Male	Reference	-	1.0		
**AGE CATEGORY**					
Geriatric	0.74 (0.19)	<0.0001	4.4	2.1	9.3
Non-geriatric	Reference	-	1.0		
**LACTATE**					
Not recorded	1.20 (0.27)	<0.0001	15	6.7	37
High	0.32 (0.27)	0.24	2.4	0.97	6.2
Low	Reference	-	1.0		

a *Interpretation: After adjusting for the effect of deposit required, sex, age category and lactate, the odds of euthanasia for a patient who was not insured was 7.4 (95% CI, 2.0 to 37) times the odds of a patient who was insured*.

### Mortality and Mode of Death During and After Surgery

The survival to discharge outcome was recorded for 257 animals (99%; 95% CI, 99% to 100%). A large proportion of dogs (*n* = 106, 41%; 95% CI, 35 to 47%) did not survive to discharge, but most non-survivors died prior to surgery (*n* = 82, 77%; 95% CI, 69 to 84%). Of the 175 dogs that underwent surgery, 151 (86%; 95% CI, 80 to 91%) survived to hospital discharge. Of the 24 animals that died despite surgical intervention, euthanasia (during or following surgery) accounted for most deaths (*n* = 19, 79%; 95% CI, 60 to 91%) compared with CPA (*n* = 3, 13%; 95% CI, 4 to 31%). For two dogs the mode of death was not recorded (8%; 95% CI, 2 to 26%).

Based on our logistic regression analyses including insurance status, lactate category, sex and geriatric denomination as explanatory variables, the odds of death for geriatric dogs undergoing surgery was 4.0 (95% CI 1.4 to 11, *p* < 0.01) times that of non-geriatric dogs. Insurance status (*p* = 0.29), sex (*p* = 0.21) and blood lactate concentration (high vs. low, *p* = 0.19) were not statistically significantly associated with survival, once the decision for surgery was made.

## Discussion

Our study demonstrated that firstly, most mortality in non-referred dogs with GDV was a result of euthanasia prior to surgery, and second, insurance markedly attenuated the occurrence of euthanasia at this time point. The first finding further validates the observation in three recent publications from three different countries that found that 23 to 38% of dogs with GDV were euthanized before surgery (9, 13, 14). We found that these animals undergoing pre-surgical euthanasia comprised three-quarters of all deaths, which parallels reports in three other studies where 65, 73, and 82% of deaths of dogs with GDV occurred due to euthanasia before treatment (9, 13, 14). Once animals undergo surgical treatment, reported survival rates are relatively good and have remained stable between 80 and 90% since 2010, with the pooled survival rate from 1,590 GDV cases being 86% (9–11, 13, 14, 20). While further improvements in perioperative care could increase this survival rate and efforts to optimize such care should be undertaken, the effect will likely be limited to a few percentage points. A much larger opportunity to reduce preventable deaths lays in flattening the early wave of mortality due to pre-surgical euthanasia.

We hypothesized that pre-operative euthanasia in non-referred dogs with GDV is first and foremost economically motivated and therefore amenable to an intervention that reduces veterinary medical cost to pet owners. We showed in our study that pet health insurance, the archetypical instrument for alleviating out-of-pocket expenses for medical care, is highly effective in mitigating pre-operative euthanasia in non-referred dogs with GDV presenting to veterinary emergency clinics. Previous studies and the collective experience in the profession suggest that other factors, notably advancing age, severity of illness (i.e., prognosis) and concurrent diseases (i.e., comorbidities) will inform dog owners' decisions to elect euthanasia over surgical treatment, independent of cost (13). Our findings suggest that age, in particular, has a significant impact on the pre-surgical euthanasia decision. This and other non-economic decision factors may explain that the group of insured animals in our study was not immune to pre-surgical euthanasia, with 1 in 10 insured dogs still undergoing humane euthanasia. These 10% of animals that were euthanized despite insurance coverage may represent the *economy-independent* portion of pre-surgical mortality. The percentage difference between euthanasia rates of insured and non-insured animals would then represent the *economy-dependent* portion of pre-surgical mortality. Consequently, ~27% of all dogs presenting for GDV and 61% of all non-survivors were euthanized for economic reasons in the emergency room.

Our study results infer that the biggest opportunity in saving lives of dogs with GDV is not medical but economic in nature. We showed that economic euthanasia can be attenuated by financial interventions, such as pet insurance. Alternate economic instruments could include payment plans, lower deposits, third-party financing options, charitable trusts or cost reduction strategies (6, 21). Prophylactic gastropexy reliably prevents unexpected out-of-pocket expenditure for emergency treatment of GDV and may be cost-effective for breeds at high risk of GDV (22).

While our study focused on treatment of dogs with GDV in emergency hospitals, the issue extends to other clinical contexts. Kipperman et al. found that amongst small animal veterinarians in the USA, 38% of respondents conducted economic euthanasia at least a few times a month, and 76% experienced compromised patient care due to financial limitations at least a few times a week (5). Similarly, Kondrup et al. reported that small animal veterinarians in Denmark regularly treat animals of financially limited clients (2, 5).

The disparity between cost of medical care and affordability constitutes a significant source of distress for pet owners as well as veterinarians above and beyond the death of the animal. For veterinarians, economic euthanasia where a viable medical alternative exists, is a major ethical dilemma and contributes to moral distress and professional burnout (1, 2, 23). This further exacerbates the effect of high workloads, low salaries, accumulated student debt, and a high compassion burden on the well-documented poor psychological health of veterinary professionals (24, 25). For pet owners, the experience of euthanasia for financial reasons while viable medical options exist is more stressful than euthanasia for futility where no such medical options are on offer, especially where strong attachment is present (3).

Our study results also emphasize that insurance status data should be collected and reported in studies with survival as a critical outcome, given the significant effect of insurance on survival of dogs with GDV and possibly other diseases such as trauma.

Several limitations require consideration when interpreting the findings. Data on control (i.e., non-insured) animals were obtained from a convenience sample of emergency practices willing to contribute to the study upon request and was not a random sample of all emergency practices. In addition, we only included animals with a non-ambiguous statement on insurance status in the EMR. Thus, only a subset of all eligible, non-insured animals was included in the study. Data for insured animals originated from a pet insurance provider that underwrites ~80% of all dog policies in Australia and will thus represent the majority of insured dogs that were eligible. Despite our study including dogs from 24 emergency hospitals in the metropolitan region of Melbourne and Sydney, dogs with GDV, whether they were insured or not, were undoubtedly seen at other veterinary facilities during the study period. Based on demographics and outcome, our study population characteristics however are comparable to those reported by others (9–11, 13, 14, 20).

Euthanized and non-euthanized animals were not perfectly matched in relevant patient characteristics that could have influenced the decision for pre-surgical euthanasia in addition to insurance. A larger data set would have been required to apply methodologies such as propensity matching to control for all observed covariates at baseline (26). Instead, we employed a logistic regression model to adjust the impact of insurance on euthanasia for obvious confounders. Given the retrospective study design, some confounders may have been missed. The socioeconomic status of pet owners, the recommendation and the estimate provided by the attending veterinarian or the impact of pre-existing comorbidities on quality of life were not captured. We may have missed relevant comorbidities as the EMR records at emergency clinics might be incomplete in this regard, and possibly more so in cases that are not treated. What the combined effect of these factors is on our study results is difficult to ascertain, as some may enhance, and others impede the decision for pre-surgical euthanasia on economic grounds. Nevertheless, integrating such data in prospective studies will reduce interpretative uncertainty in the future.

Study inclusion required radiographic confirmation of GDV cases to ensure validity of subsequent outcomes (8). Notably, this requirement may have preferentially excluded some dogs in the pre-surgical euthanasia group due to the cost associated with radiographs and thus led to an underestimate of economic euthanasia. We found a strong association between the lack of a recorded lactate level and pre-surgical euthanasia, and the absence of radiographs may have had a similar effect.

Finally, pet insurance did not entirely negate the need for out-of-pocket expenses. Foremost, all participating hospitals required an identical deposit, typically around 50% of the estimate or several thousand dollars, whether insurance was present or not and the pet owner was liable for that deposit at admission of the animal. A “gap-only” or “co-pay” payment option for insured animals was not available at the time of the study. Moreover, most insurance policies do not cover the entire veterinary bill, such that an out-of-pocket expense remains even for pet owners with insurance. It is therefore reasonable to assume that these factors may have contributed to the decision of pre-surgical euthanasia of insured animals.

In summary, this multicenter retrospective study showed that most non-survivors among non-referred dogs with GDV presented to emergency hospitals die prior to surgery by means of humane euthanasia, and we have provided evidence that the decision for euthanasia is predominantly economic in origin. In animals presenting with life-threatening but treatable disease, financial interventions to reduce economic euthanasia therefore constitute an essential opportunity to mitigate preventable deaths and optimize system wide patient outcomes.

## Data Availability Statement

The datasets generated for this study are available on request to the corresponding author.

## Author Contributions

MB designed the study, developed the research database, analyzed and interpreted data, and wrote the manuscript. TN and JA contributed to the study design, data collection and writing of the manuscript. MA and JS contributed to study design and data collection, but not the data analysis or interpretation of the data. MS contributed to the study design and analyzed and interpreted the data. EB contributed to the writing of the manuscript. All authors critically revised and approved the manuscript.

## Conflict of Interest

MA and JS were employed by the company PetSure, an Australian pet insurance provider. The remaining authors declare that the research was conducted in the absence of any commercial or financial relationships that could be construed as a potential conflict of interest.

## Abbreviations

CPA: Cardiopulmonary arrest
GDV: Gastric dilatation-volvulus
ECC: Emergency and critical care
NSW: New South Wales
VIC: Victoria.
