# Supplementary material for: The Effect of Pet Insurance on Presurgical Euthanasia of Dogs With Gastric Dilatation-Volvulus: A Novel Approach to Quantifying Economic Euthanasia in Veterinary Emergency Medicine
Source: Front Vet Sci. 2020 Dec 8;7:590615. doi: 10.3389/fvets.2020.590615 (PMC7752994; doi:10.3389/fvets.2020.590615)
Supplement: Supplementary file 1 [file Data_Sheet_1.PDF]

| | | | |
|---|---|---|---|
| Record ID | text, Required | | |
| 2 | hospital_id | Section Header: *Hospital information*<br>Hospital ID | text |
| 3 | state_hospital | State in which hospital is located: | radio, Required, Identifier<br>1 VIC<br>2 NSW<br>3 SA<br>4 QLD<br>5 WA |
| 4 | postcode_hospital | Post code of hospital: | text (postalcode_australia), Identifier |
| 5 | hospital_setting | Which of the following best matches the hospital setting? | radio, Required<br>1 General practitioner practice<br>2 Referral practice with specialists |
| 6 | emergency_type | Which of the following best describes the emergency service the hospital provides? | radio, Required<br>1 Emergency service during regular business hours only<br>2 Emergency service during regular business hours and on call service after hours<br>3 Emergency clinic or service open after regular business hours only<br>4 Emergency clinic or service open 24hrs and able to hospitalize patients<br>5 Emergency/critical care center with at least one board-certified emergency clinician<br>6 Other |
| 7 | dep_nonins | Is a deposit required for NON-insured cases? | radio, Required<br>1 Yes<br>2 No<br>3 Unsure/Unknown |
| 8 | deposit_policy<br>Show the field ONLY if:<br>[dep_nonins] = '1' | What is the deposit requirement? | radio, Required<br>1 25% of HIGHER end of quotation<br>2 50% of HIGHER end of quotation<br>3 The entire quoted amount<br>4 25% of LOWER end of quotation<br>5 50% of LOWER end of quotation |

| | | | |
|---|---|---|---|
| | | | 6 Other |
| 9 | deposit_policy_other<br>Show the field ONLY if:<br>[deposit_policy] = '6' | What other deposit? | text, Required |
| 10 | dep_insur | Is a deposit required for insured cases? | radio, Required<br>1 Yes<br>2 No<br>3 Unsure/Unknown |
| 11 | deposit_insured_pol<br>Show the field ONLY if:<br>[dep_insur] = '1' | Is this deposit the same as for non-insured cases? | radio, Required<br>1 Yes, it is the same<br>2 No, it is LOWER<br>3 No, it is HIGHER |
| 12 | vetpay | Is this hospital offering VetPay? | radio, Required<br>1 Yes<br>2 No<br>3 Unsure/Unknown |
| 13 | deposit_vetpay<br>Show the field ONLY if:<br>[vetpay] = '1' | What is the deposit required when choosing VetPay (% of quotation)?<br>*Enter the percentage that applies to a GDV quote (> 5000 AUD)* | text (integer), Required |
| 14 | payment_plan | Is this hospital offering payment plans? | radio, Required<br>1 Yes<br>2 No<br>3 Unsure/Unknown |
| 15 | deposit_pp<br>Show the field ONLY if:<br>[payment_plan] = '1' | What is the deposit required when choosing a payment plan (% of quotation)?<br>*Enter the percentage that applies to a GDV quote (> 5000 AUD)* | text (integer), Required |
| 16 | hospital_information_complete | Section Header: *Form Status*<br>Complete? | dropdown<br>0 Incomplete<br>1 Unverified<br>2 Complete |
