# Supplementary material for: The Effect of Pet Insurance on Presurgical Euthanasia of Dogs With Gastric Dilatation-Volvulus: A Novel Approach to Quantifying Economic Euthanasia in Veterinary Emergency Medicine
Source: Front Vet Sci. 2020 Dec 8;7:590615. doi: 10.3389/fvets.2020.590615 (PMC7752994; doi:10.3389/fvets.2020.590615)
Supplement: Supplementary file 2 [file Data_Sheet_2.PDF]

record_id	Record ID	text, Required	
2	hospital_id	Section Header: *Animal information*<br>Hospital ID	text, Required
3	animal_id	Animal ID (e.g., MRN)	text, Required
4	insurance	Insurance present at time of GDV?	radio, Required<br>0 Yes<br>1 No<br>2 Unknown/Not recorded
5	referred	Was the animal referred?	radio, Required<br>0 Yes<br>1 No<br>2 Unknown/Not recorded
6	species	Species	radio, Required<br>0 Dog<br>1 Cat
7	pure_bred	What is the breed of the dog?	radio, Required<br>1 Mixed breed<br>2 Pure breed<br>3 Not sure/unknown
8	breed	Which breed?	dropdown, Required<br>1 Affenpinscher<br>2 Afghan Hound<br>3 Airedale Terrier<br>4 Alaskan Malamute<br>5 American Staffordshire Terrier<br>6 Anatolian Shepherd Dog<br>7 Australian Cattle Dog<br>169 Australian Kelpie<br>8 Australian Shepherd<br>9 Australian Silky Terrier<br>10 Australian Terrier<br>11 Basenji<br>12 Basset Fauve De Bretagne<br>13 Basset Hound<br>14 Beagle

			15 Bearded Collie<br>16 Bedlington Terrier<br>17 Belgian Shepherd Dog<br>18 Bernese Mountain Dog<br>19 Bichon Frise<br>20 Bloodhound<br>21 Border Collie<br>22 Border Terrier<br>23 Borzoi<br>24 Boston Terrier<br>25 Bouvier Des Flandres<br>26 Boxer<br>27 Briard<br>28 Brittany<br>29 Bull Terrier<br>30 Bull Terrier<br>31 Bulldog<br>32 Bullmastiff<br>33 Cairn Terrier<br>34 Canaan Dog<br>35 Cavalier King Charles Spaniel<br>36 Chesapeake Bay Retriever<br>37 Chihuahua<br>38 Chinese Crested Dog<br>39 Chow Chow<br>40 Collie<br>41 Curly Coated Retriever<br>42 Dachsbrake<br>43 Dachshund (Long-Haired)<br>44 Dachshund (Miniature Long-Haired)<br>45 Dachshund (Miniature Smooth-Haired)<br>46 Dachshund (Miniature Wire-Haired)<br>47 Dachshund (Smooth-Haired)<br>48 Dachshund (Wire-Haired)

			49 Dalmatian<br>50 Dandie Dinmont Terrier<br>51 Deerhound<br>52 Dobermann<br>53 Elkhound<br>54 English Setter<br>55 English Toy Terrier<br>56 Eskimo Dog<br>57 Estrela Mountain Dog<br>58 Finnish Spitz<br>59 Flat Coat Retriever<br>60 Fox Terrier (Smooth)<br>61 Fox Terrier (Wire)<br>62 Foxhound<br>63 French Bulldog<br>64 German Longhaired Pointer<br>65 German Shepherd Dog<br>66 German Shorthaired Pointer<br>67 German Spitz<br>68 German Wirehaired Pointer<br>69 Giant Schnauzer<br>70 Glen of Imaal Terrier<br>71 Golden Retriever<br>72 Gordon Setter<br>73 Great Dane<br>74 Greyhound<br>75 Griffon Bruxellois<br>76 Hamiltonstovare<br>77 Harrier<br>78 Hovawart<br>79 Hungarian Kuvasz<br>80 Hungarian Puli<br>81 Hungarian Vizla<br>82 Hungarian Wire Haired Vizla<br>83 Ibizan Hound<br>84 Iceland Dog<br>85 Irish Red and White Setter

			86 Irish Setter<br>87 Irish Terrier<br>88 Irish Wolfhound<br>89 Italian Greyhound<br>90 Italian Spinone<br>91 Jack Russell Terrier<br>92 Japanese Akita<br>93 Japanese Chin<br>94 Japanese Shiba Inu<br>95 Japanese Spitz<br>96 Keeshond<br>97 Kerry Blue Terrier<br>98 King Charles Spaniel<br>99 Komondor<br>100 Labrador Retriever<br>101 Lakeland Terrier<br>102 Lancashire Heeler<br>103 Large Munsterlander<br>104 Leonberger<br>105 Lhasa Apso<br>106 Lowchen (Little Lion Dog)<br>107 Maltese<br>108 Manchester Terrier<br>109 Maremma Sheepdog<br>110 Mastiff<br>111 Mexican Hairless<br>112 Miniature Pinscher<br>113 Neapolitan Mastiff<br>114 Newfoundland<br>115 Norfolk Terrier<br>116 Norwegian Buhund<br>117 Norwich Terrier<br>118 Old English Sheepdog<br>119 Otterhound<br>120 Papillon<br>121 Pekingese<br>122 Petit Basset Griffon Vendeen

			123 Pharaoh Hound<br>124 Pinscher<br>125 Pointer<br>126 Polish Lowland Sheepdog<br>127 Pomeranian<br>128 Poodle (Miniature)<br>129 Poodle (Standard)<br>130 Poodle (Toy)<br>131 Portuguese Water Dog<br>132 Pug<br>133 Pyrenean Mountain Dog<br>134 Rhodesian Ridgeback<br>135 Rottweiler<br>136 Saluki<br>137 Samoyed<br>138 Schipperke<br>139 Schnauzer<br>140 Schnauzer (Miniature)<br>141 Scottish Terrier<br>142 Sealyham Terrier<br>143 Setter Red and White<br>144 Shar Pei<br>145 Shetland Sheepdog<br>146 Shih Tzu<br>147 Siberian Husky<br>148 Skye Terrier<br>149 Sloughi<br>150 Soft Coated Wheaten Terrier<br>151 Spaniel (American Cocker)<br>152 Spaniel (American Water)<br>153 Spaniel (Clumber)<br>154 Spaniel (Cocker)<br>155 St. Bernard<br>156 Staffordshire Bull Terrier<br>157 Swedish Vallhund<br>158 Swiss Laufhund<br>159 Thai Ridgeback<br>160 Tibetan Mastiff

			161 Tibetan Terrier<br>162 Weimaraner<br>163 Welsh Corgi<br>164 Welsh Terrier<br>165 West Highland White Terrier<br>166 Whippet<br>167 Yorkshire Terrier<br>168 Other
9	other_breed<br>Show the field ONLY if:<br>[breed] = '168'	Which other breed?	text
10	sex	Sex	radio, Required<br>0 Female intact<br>2 Female spayed<br>5 Female, desexing status unknown/not recorded<br>1 Male intact<br>3 Male neutered<br>6 Male, desexing status unknown/not recorded<br>4 Unknown/Not reported
11	age	Age at time of event<br>*Full years if > 1 year; decimal if < 1 year*	text (number, Max: 20), Required
12	preexisting_cond	Any comorbid condition?	radio, Required<br>1 Yes<br>2 Unknown/Not recorded
13	comorbid_cond<br>Show the field ONLY if:<br>[preexisting_cond] = '1'	List comorbid conditions:	notes, Required
14	date_event	Section Header: *Event variables*<br>Date of presentation to hospital<br>*DD-MM-YYYY*	text (date_dmy), Required
15	diagnostics	What diagnostics were performed?	checkbox, Required<br>0 diagnostics___0 Physical exam (PE) only

			1 diagnostics___1 Blood work<br>3 diagnostics___3 Abdominal Radiographs
16	gdv_confirmed	Was the GDV diagnosis confirmed by radiographs?	radio, Required<br>1 Yes<br>2 No<br>3 Unsure/Unknown
17	lactate_done	Was lactate at admission measured?	radio, Required<br>1 Yes<br>2 No<br>3 Unsure/Unknown
18	lactate	Lacate (mmol/L) at presentation	text (number, Min: 0.2, Max: 20)
19	euth_before	Section Header: *Animal outcome*<br>Euthanasia before surgical treatment	radio, Required<br>0 Yes<br>1 No<br>2 Unknown/Not recorded
20	outcome_surgery<br>Show the field ONLY if:<br>[euth_before] = '1'	Outcome if surgery initiated<br>*Outcome after treatment initiated!*	radio, Required<br>1 Survived to hospital discharge<br>2 Died: euthanasia<br>3 Died: arrested<br>4 Died: unknown/not recorded<br>5 Unknown/not recorded
21	animal_event_and_outcome_complete	Section Header: *Form Status*<br>Complete?	dropdown<br>0 Incomplete<br>1 Unverified<br>2 Complete
